# Exploration of an Alarm Sensor to Detect Infusion Failure Administered by Syringe Pumps

**DOI:** 10.3390/diagnostics12040936

**Published:** 2022-04-08

**Authors:** Florian Wieduwilt, Jasmin Grünewald, Georgios Ctistis, Christoph Lenth, Thorsten Perl, Hainer Wackerbarth

**Affiliations:** 1 Institut für Nanophotonik Göttingen e.V., Hans-Adolf-Krebs-Weg 1, 37077 Göttingen, Germany; jasmin.gruenewald@ifnano.de (J.G.); christoph.lenth@ifnano.de (C.L.); hainer.wackerbarth@ifnano.de (H.W.); 2 Physical Chemistry of Nanomaterials, Institute of Chemistry and Center for Interdisciplinary Nanostructure Science and Technology (CINSaT), University of Kassel, Heinrich-Plett-Straße 40, 34132 Kassel, Germany; 3 Department of General, Visceral and Pediatric Surgery, University Medical Center Göttingen, Robert-Koch-Straße 40, 37075 Göttingen, Germany; tperl@medizin.uni-goettingen.de

**Keywords:** patient safety, point-of-care diagnostics, infusion monitoring, medication errors, drug monitoring, refractive index, UV-Vis, spectrophotometry

## Abstract

Incorrect medication administration causes millions of undesirable complications worldwide every year. The problem is severe and there are many control systems in the market, yet the exact molecular composition of the solution is not monitored. Here, we propose an alarm sensor based on UV-Vis spectroscopy and refractometry. Both methods are non-invasive and non-destructive as they utilize visible light for the analysis. Moreover, they can be used for on-site or point-of-care diagnosis. UV-Vis-spectrometer detect the absorption of light caused by an electronic transition in an atom or molecule. In contrast a refractometer measures the extent of light refraction as part of a refractive index of transparent substances. Both methods can be used for quantification of dissolved analytes in transparent substances. We show that a sensor combining both methods is capable to discern most standard medications that are used in intensive care medicine. Furthermore, an integration of the alarm sensor in already existing monitoring systems is possible.

## 1. Introduction

Incorrect medication administration causes millions of undesirable complications worldwide every year. This might be a consequence of the accuracy and quality of the documentation and the personal assigned with the reporting of these incidents [1,2,3,4,5]. It must be assumed that only a small proportion of incorrect medications is officially reported or recognized as such. In a meta-analysis of preventable adverse drug reactions by Hakkarainen et al. [6], eight studies with 24.128 patients were considered. Among inpatients in five different countries, 1.6% had preventable adverse drug reactions and 45% of adverse drug reactions were preventable, each with a confidence interval of 95%. The results of the eight studies, however, range between 0.1 and 51% of preventable adverse drug reactions [7,8,9,10,11,12,13,14].

Modern intensive care therapy includes pharmacological treatment with various drugs that must be administered intravenously continuously. Further development of therapeutic treatment and the associated increasing complexity of infusion therapy in intensive care medicine poses challenges and problems for both medical and nursing staff, which ultimately increases the risk of adverse medication errors [15,16]. In addition, traditionally handwritten documents are a non-negligible source of subsequent medication errors [17,18]. Incorrect medications, especially in complex intensive care, are therefore inherent in the system due to human errors such as mix-ups of medications or false patient assignment and are cited in the literature as approximately 5% of medications [19]. The consequences of incorrect medication can range up to a vital threat to the patient and thus pose a risk to patient safety. Medication errors occur more frequently in intensive care units (ICU) settings due to multiple medications per case, specific and complex dose regimes for the used medications and critical conditions of patients with organ insufficiency, dynamic changes, and associated difficult dynamic situations in critical care medicine [20]. According to a CDC ranking, medical errors are the third most common cause of death in the US [21]. The significance and extent of the damage becomes clear when considering that more than 600 million intravenous infusions are administered to patients in Europe alone each year.

Intravenous medications are used to administer high-risk drugs which can have serious consequences for the patient if incorrectly administered. To ensure patient safety and to reduce medication errors, intelligent infusion systems are increasingly being used in hospitals, although their application entails new challenges as well as many positive aspects [22,23]. In addition to a user-friendly interface, intelligent infusion pumps also have a dose error reduction system (DERS), which can use a database to report errors when setting the dose [24]. The Bar Code Medication Administration (BCMA) system is also used. These are barcodes that are attached to the bag/container of the infusion solution. In addition, the patient receives a bracelet with a personal barcode. Before administration of a drug with an intravenous infusion, the barcodes of the infusion solution and the patient’s wristband are scanned to avoid mix-ups [25]. There are also various options for monitoring the flow rate. One example is the process of drop detection and counting to determine the flow rate using a camera and object recognition software [26]. Another approach is a drip bag scale, which can deduce the flow rate due to the reduced weight of the infusion bag [27]. Additionally, there are pressure sensing techniques to detect occlusions in infusion tubing [28]. Intelligent infusion pumps and the methods presented can reduce the number of medication errors, but not completely eliminate them [24]. Current studies reveal that 67% of administered intravenous infusions show deviations from the original prescription [29]. The most common sources of error are: not administering an infusion (22.9%), an incorrect flow rate (20.3%), an incorrectly administered drug (16.9%), and an incorrect dose (13.6%) [24]. Further studies show that the cause of medication errors is less caused by malfunctions in the software of the infusion pumps than by operating errors and a lack of use of safety systems [29,30]. The current state of research offers various solutions to reduce errors caused by an incorrect flow rate and solutions to support staff in avoiding operating errors. Available systems in research as well as industry do not yet offer any substance specific electronic monitoring, that allows the contents of an infusion fluid and its concentration to be checked for accuracy. The BCMA system represents the first approach in this direction, whereby the system’s dependency on the code attached to the infusion bag must be noted.

In the present article we investigate suitable monitoring techniques that can be integrated in intravenous therapy. The intravenous therapy delivers solutions such as fluids, medications, and nutrition directly into a patient’s vein. Syringe pumps or infusion pumps are used for the delivery. Solutions are fluid homogenous mixtures of a least two substances. In the case of a medical infusion, the solvent is mostly water, although there are exceptions such as lipid infusions (oil in water emulsions) for nutrition.

In principle, monitoring techniques for solutions can be separated into two types. The first one reflects the physical property of the entire solution such as refractive index, density, and conductivity. Techniques to measure these properties are refractometry, ultrasound velocimetry, as well as electrochemical conductometry and impedance. Techniques of the second type can directly identify the molecules of the solutions. Here, nuclear magnetic resonance (NMR), Raman scattering, IR- and UV-Vis absorbance, and fluorescence are the most viable ones. NMR is a quite costly and sophisticated approach, which is not suitable for point-of-care monitoring. Near- and mid-IR spectroscopy are in principle available as point-of-care devices, but due to the presence of water, which is one of the strongest absorbers, the signatures of the other molecules are masked [31]. Raman spectroscopy, on the other hand, is complimentary to the IR spectroscopy and provides the fingerprint of molecules in aqueous solutions, since it is not affected by water. There are already point of care devices available [32,33,34]. The same is true for both UV-Vis spectrophotometry and fluorescence spectroscopy. However, these techniques do not provide a spectroscopic fingerprint of the molecule, they provide information of the fluorophore and the chromophore, respectively. Therefore, these techniques allow a broad estimation of chemical substance classes.

Here, the abilities of two technical approaches for direct monitoring of intravenous infusions by the determination of physical parameters are demonstrated for the first time. The recognition of frequently used solutions and medications in intensive care medicine is therefore possible. The physical parameters are the refractive index and the UV-Vis absorption, which can identify both, the type of infusion and also its concentration. This could reduce the error rates for the administration or wrong medications and wrong dosages through a substance-specific analysis of the medications used.

## 2. Materials and Methods

In cooperation with the University Medical Center (UMC) Gottingen, frequently and continuously used medications and (carrier-) fluids were selected. For this study, we selected the following medical solutions applied in the daily practice of UMC Göttingen intensive care unit: catecholamine (norepinephrine and epinephrine), an antibiotic drug (vancomycine), and insulin. These medications are prepared in water-based solution as carrier fluid. As carrier fluid glucose and sodium chloride solution are applied and were investigated additionally. Further electrolyte formulations in different clinically relevant and commercially available concentrations (sodium chloride, potassium chloride, and sodium bicarbonate (NaHCO_3_)) were investigated.

The ability to distinguish and recognize crystalloid infusion solutions, such as sodium chloride and potassium chloride solutions, is crucial. For instance, for a patient who suffers from diabetes and needs urgently medication, a confusion of insulin and potassium chloride infusion will end up being fatal. Some solutions and mixtures were investigated which are not used in this form in hospitals but help to better estimate the possible range of values and the influence of the carrier substance on the physical properties. Table 1 and Table 2 list the solutions and mixtures prepared and used for the experiments.

Aqua, glucose 5% and 40%, sodium chloride 0.9%, 5.85%, and 20%, potassium chloride 7.45% and 20%, and sodium bicarbonate 8.4% were purchased from B. Braun Melsungen AG, Germany. Norepinephrine as Arterenol, epinephrine as Suprarenin, Vancomycin, and insulin as Insuman were obtained from Sanofi, Germany. Additionally, deionized water (Millipore 18 MΩ) was used. It is important to note that in our study, deionized water and aqua had the same refractive index and were therefore indistinguishable. The dispersion refractometer ATR-L was purchased from the company Schmidt + Haensch, Germany, which performs the calculation of the refractive index by means of total reflection. An accuracy of $\pm \;4\times 10^{-5}$ with a precision of the Peltier temperature control of the prism of ΔT=0.03 °C is provided. The refractive indices were determined at a wavelength of 589 nm. The spectrophotometer, UV-1900i from Shimadzu, Germany, enables absorption analysis in the measurement wavelength range from 190 to 1100 nm. The resolution of the device is 0.05 nm with a wavelength accuracy of ±0.1nm. Photometric accuracy decreases with increasing absorbance and ranges from ±0.002 to ±0.006 arbitrary absorbance units. The wavelengths were recorded with a wavelength interval of 0.2 nm and an average speed of about 60nm/min.

## 3. Results

### 3.1. Refractometry

One of the most important quantities describing a material is its refractive index, i.e., its ability to interact with light, given by $n=c_{0}⁄c_{m}$, where $c_{0}$ is the speed of light in vacuum and $c_{m}$ the speed of light in the material, respectively. Since the refractive index originates from the oscillator strength inside the material, thus being a material property, it strongly depends on the temperature but in the case of solutions also on the concentration of the material. For not too extreme conditions, the refractive index changes linearly with concentration [35]. The refractive index can easily be measured with a refractometer, where the refracting angle of a light beam passing through a material is accurately measured. With this information, the quantification of binary solutions is possible. However, since the refractive index is not uniquely linked to a single material, it is not possible to distinguish between materials with the same refractive index based on this information alone.

**Figure 1:**
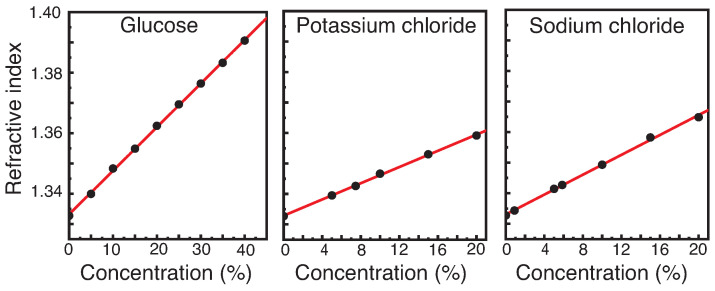
Refractive indices measured at a wavelength of λ=589nm and a temperature of T=23 °C for solutions with varying concentration of glucose, potassium chloride, and sodium chloride. The fits show a linear dependence between refractive index and concentration.

The values of the experimentally determined refractive indices for different concentrations of solutions of aqueous glucose, sodium chloride (NaCl), and potassium chloride (KCl) are shown in Figure 1. As expected, all three solutions show a linear correlation of refractive index with concentration, i.e., the higher the concentrations, the higher the refractive index becomes. The extracted values for the slope and the intercept with the ordinate are listed in Table 3. The errors for the refractive index and the corresponding concentration are so small in this graphical representation that they disappear under the data points in the selected plot.

From Figure 1 it becomes apparent that the refractive index on its own is not suitable for the unambiguous identification of solutions. For example, a refractive index of 1.34000 corresponds to a NaCl-solution of 4.2%, a KCl-solution of 5.3%, as well as a glucose solution of 4.8%. However, when administering infusions in medical care, only specific (standardized) concentrations of the solutions are used. For example, NaCl is used only in concentrations of 0.9%, 5.85%, 10%, and 20%, KCl in concentrations of 7.45%, 14.9%, and 20%, and glucose in concentrations of 5%, 10%, 20%, and 40%. Therefore, in addition to the type of infusion, their usual concentration was also taken into account in the selection.

**Figure 2:**
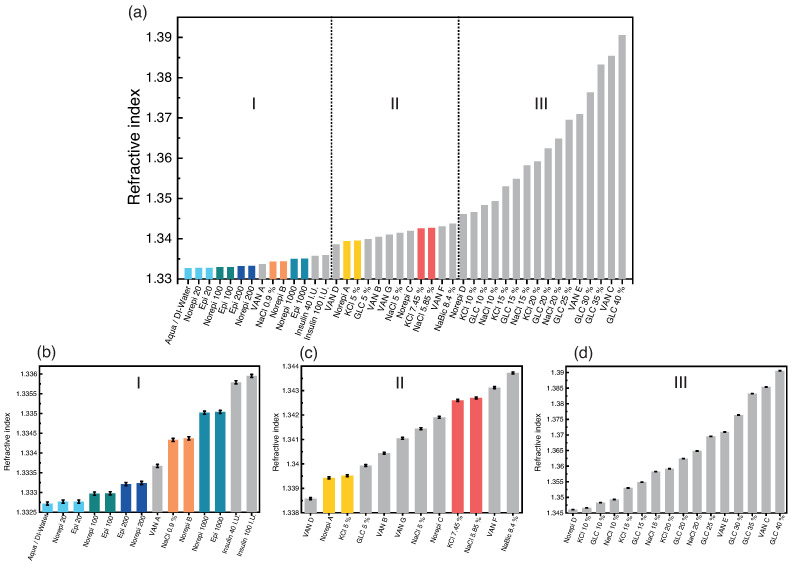
(**a**) Refractive index measured at a wavelength of λ=589nm and a temperature of T=23 °C for different solutions and concentrations (for abbreviations of the solutions, see Table 1 and Table 2). (**b**) Magnification of the refractive indices of the first segment of panel (**a**) with error bars. (**c**) Magnification of the refractive indices of the second segment of panel (**a**) with error bars. (**d**) Magnification of the refractive indices of the third segment of panel (**a**) with error bars.

Figure 2a shows the refractive indices of a series of 42 solutions and medications being used in medical care, divided into segment I, II, and III. The refractive index hereby ranges from 1.33273 (aqua) to 1.39057 (GLC 40%), respectively. As expected, the high refractive indices (n>1.34400) in segment III belong to solutions of high concentrations. These 16 solutions can be distinguished solely by the refractive indices, as their values are clearly different. This is depicted in Figure 2d. Due to the large differences in the refractive indices, the error margins are not significant and are also negligible. Compared to the refractive index values, they are so small that they can only be recognized as black lines on the value bar. The remaining 26 solutions show a refractive index between 1.3373 and 1.34400, resulting in a higher density of values in this range. Among these 26 medications and solutions of segment I and II, there are seven pairs that cannot be clearly distinguished by the refractive index. These include the pairs of epinephrine and norepinephrine at concentrations of 20, 100, 200, and 1000μg/mL since the molecular structures of the catecholamines are very similar which is shown in Figure 4 (vide infra).In the case of 20μg/mL, the two mixtures are so diluted that their refractive indices cannot even be distinguished from deionized water or Aqua. The remaining three pairs are NaCl 0.9%/Norepi B, Norepi A/KCl 5%, and KCl 7.45%/NaCl 5.85%.

**Figure 3:**
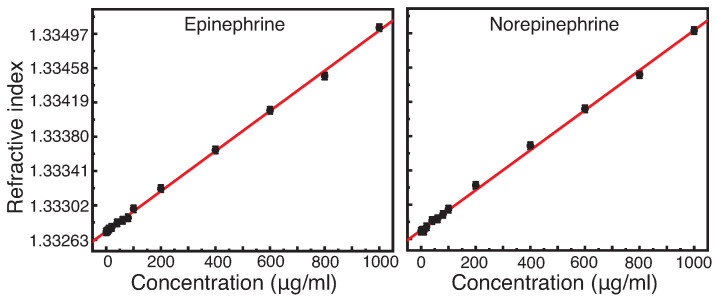
Refractive index measurement at a wavelength of λ=589nm and a temperature of T=23 °C for varying concentrations of norepinephrine and epinephrine within a concentration range of 0 and 1000μg/mL.

For verification of the exact limit of detection (LOD) and limit of quantification (LOQ) of the catecholamines, solutions are probed by means of refractometry. In Figure 3, the refractive indices of usual doses of norepinephrine and epinephrine are plotted against their concentrations. The errors for the depicted catecholamine concentrations are in the range of 1% or less in our investigations under laboratory conditions. In this order of magnitude (≤1%), the error bars are masked by the actual data points. In Figure S1 of the supplementary material, an enlargement of the area between 0 and 100μg/mL is shown, where the error bars become visible. The errors that might occur during the production of solutions like these in everyday medical use cannot be estimated on the basis of our laboratory experience, but are supposed to be above our error limits.

In Figure 3, a linear correlation between the concentration of norepinephrine and epinephrine and the refractive index can be observed with a slope of $2.27\left(2\right)\times 10^{-6}$ and $2.28\left(2\right)\times 10^{-6}$, respectively (see also Table 4). Analysing the refractive index measurements further, the LOD and LOQ could be determined. Their respective values are listed in Table 5.

These results clearly show that by refractive index measurements a dose of 60μg/mL, which is most frequent administered in hospitals, can be detected but cannot be quantified. Based on the previous refractive index measurements, it was expected that similar LODs and LOQs would be found for epinephrine and norepinephrine. The deviations of the limit values from each other therefore probably originate from the errors in the preparation of the different concentrations and the subsequent linear regression. Hence, in the case of the low concentrations, a weakness of the technique is shown. Therefore and due to the fact that some medications are indistinguishable, an additional technique is necessary. The technique must be capable of the detection of low concentrations, e.g., of catecholamines, and a differentiation of the solution by chemical structure.

### 3.2. UV-Vis Spectroscopy

Raman spectroscopy at 532 nm, 638 nm, and 785 nm was applied to the solutions, despite the low quantum efficiency of the inelastic scattering process and the resulting fact that Raman is not particularly suited for low concentrations. However, on the one hand, the optical components of Raman equipment have improved tremendously over the last twenty years and it has been established as point-of-care or on-site approach, in particular by applying surface enhanced Raman scattering [36,37]. On the other hand, the limit of detection is hard to estimate as the scattering efficiency depends on the molecular structure. Raman spectroscopy is sensitive to vibrations involving polarization changes rather than dipole moment changes and therefore shows a low scattering cross section for water [38,39]. The same applies for the polar glucose bonds providing rather weak signals while molecules with non-polar carbon bonds and aromatic systems, e.g., catecholamines, often show strong signals due to their polarizability. All aqueous solutions containing molecular components provide a fingerprint Raman spectrum, except the low concentrations of insulin and the catecholamines, which could not be identified by using reasonable recording time and laser power.

To solve the challenge of monitoring low concentration of infusions, a spectrophotometer is used, to measure absorption spectra in the UV-Vis range. The technique is fully matured and is routinely implemented in laboratories and also in different point-of-care applications with increasing share [40]. Absorption in the UV-Vis spectral range, i.e., between 200 and 800 nm is mainly caused by electronic transition of valence electrons. The absorption thereby reflects the electronic transition of an electron from a high occupied molecular orbital (HOMO) to a low unoccupied molecular orbital (LUMO) of a molecule. The behaviour of the bonding and non-bonding electrons determines the wavelength of the absorption of the molecules, enabling a differentiation between classes of substances, e.g., alkene, alkyne, carbonyl, and carboxyl compounds. Moreover, the interaction of the molecule with its environment, like the solvent, influences the absorption. The main absorption bands of pure water and aqueous sodium chloride solution are below 180 nm and thus do not interfere with the signals of the solved compounds. Thus, the measured absorbance in the visible region stems from the dissolved components.

**Figure 4:**
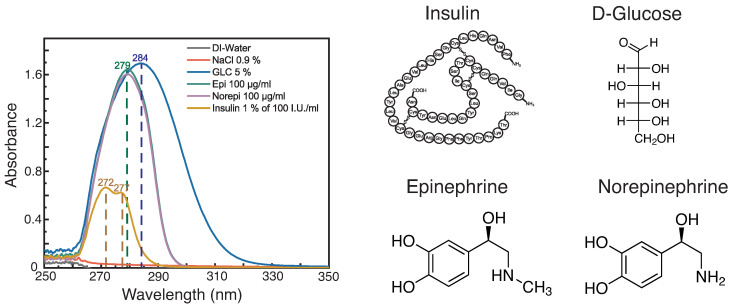
Left: Absorbance of medical solutions in their common concentrations: glucose 5% solution (blue), norepinephrine 100μg/mL (green), epinephrine 100μg/mL (violet), 1% of insulin 100 I.U. (yellow), 0.9% sodium chloride solution (red), deionized water (grey). Right: Chemical structure of insulin, glucose, epinephrine, and norepinephrine.

UV-Vis absorbance spectra of several infusions and the chemical structure of their active agents are depicted in Figure 4.

The absorbance of glucose with the maximum of 284 nm arises from the transition of $n\to \;\pi^{*}$ of the carbonyl group, while for norepinephrine and epinephrine the main absorbance is located at 279 nm. The latter are caused by a $\pi \;\to \;\pi^{*}$ transition of the aromatic structures of the active agents epinephrine and norepinephrine. Besides the active agents, norepinephrine and epinephrine contain stabilizers, which do not show any absorbance in this spectral range. A disadvantage is that the small difference in the molecular structure is not reflected through the absorbance spectrum, as the signal is caused by the aromatic ring and the associated functional groups. Thus, epinephrine and norepinephrine cannot be differentiated by absorbance. However, in contrast to the refractometry the absorbance provides direct information of the compounds of the infusions. Insulin is a protein with several chromophores including the peptide bonds, disulphides, and the aromatic amino acids. The absorbance of peptide bonds is located in the near-UV range at 190 nm, while absorbance signals from aromatic amino acids, here in particular from tyrosine and disulfides, are found in the regime of 260 to 280 nm, causing the broad absorbance with two maxima at 272 and 277 nm [41].

The strength of the UV-Vis spectrometry is the quantitative analysis. One measures thereby the intensity loss of light passing through the solution to be analyzed. In a good approximation the Bouguer–Beer–Lambert law can be used to derive the concentration from the measurement, assuming the concentration is not too high and there are no higher-order effects such as multiple scattering and re-absorption [42].

(1) $$A=\log\frac{I}{I_{0}}=-\varepsilon \cdot c\cdot d,$$

with *c* being the concentration, *I* the measured intensity, $I_{0}$ the incoming intensity, and *d* the thickness of the sample and where ε is the molar attenuation coefficient, which depends itself on the refractive index of the material.

**Figure 5:**
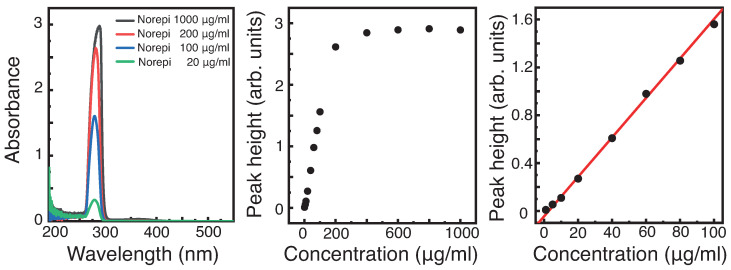
Left: Absorbance spectra of norepinephrine with varying concentrations. Middle: Extracted peak-heights from the spectrum versus a large concentration range. Right: Magnification of the low-concentration region of the middle panel showing a linear behaviour of the concentration to the absorbance.

In Figure 5, the peak height of the absorbance is plotted versus the concentration. At low concentrations, we indeed observe a linear behavior, as expected from Equation (1), between concentration and absorbance. The extracted values for the slope and the intercept with the ordinate are listed in Table 6. At approximately 200 µg/mL, no further increase is visible, indicating that the absorbance is in saturation, where the entire light is absorbed by the epinephrine. We found a similar behavior for norepinephrine. The LOD and LOQ are shown in Table 7. A similar behavior is observed when considering the area under the peak. The latter analysis should be more robust against small deviations. The comparison is shown in Figure S2 in the supplementary information.

The LOD and LOQ clearly indicate that the UV-Vis spectrometry is suited for recognition of the catecholamines at the necessary concentration. A further strong advantage is that this technique provides direct information of the substances and thus allows a differentiation.

Considering that 71% of the investigated infusion refractive indices lie between n=1.33273 and n=1.35000 and the large quantity and variety of infusions administered by syringe pumps including different concentrations, there might be overlapping of refractive indices, particularly using a point-of-care refractometer with a lower resolution. When analyzing the refractive indices of our series of experiments, seven pairs of refractive indices were identified that could not be differentiated from each other. Here, epinephrine and norepinephrine could not be distinguished at identical concentrations in each case, as well as the pairs of NaCl 0.9%/Norepi B, Norepi A/KCl 5%, and KCl 7.45%/NaCl 5.85%.

By a combination of refractive index measurements and UV-Vis spectrometry paired with intelligent data processing, it will be also possible to distinguish the pairs of NaCl 0.9%/Norepi B and Norepi A/KCl 5% from another. Thus, only the pair of KCl 7.45%/ NaCl 5.85% would remain non-distinguishable. In fact, however, the combination of these two electrolyte solutions is very unlikely to occur at these concentrations, since KCl 7.45% is usually administered with a NaCl solution of 0.9%. KCl 7.45% and NaCl 0.9% can be distinguished without any doubt.

In summary, by a combination of refractive index and spectrophotometer, most of the prepared 42 solutions could be distinguished besides the catecholamines norepinephrine and epinephrine, which have a very similar molecular structure, as well as KCl 7.45% and NaCl 5.85% solutions.

## 4. Conclusions and Outlook

Incorrect medication administration causes undesirable complications in hospitals worldwide every year. Although the problem is severe, currently there does not exist any sound technical solution to monitor the administered infusions. The challenge in monitoring infusions is thereby the large variety of generally applied solutions. On the one hand, there are the molecular substances like antibiotics, insulin, and catecholamines. The latter medications, however, are of uttermost importance at intensive care units and should therefore be addressable. A detection range of 20 µg/mL for the catecholamines is targeted. On the other hand, there are electrolytes and crystalloid infusions, such as sodium chloride and potassium chloride solutions, which are purely administered or act as carrier solutions.

Here, we present for the first time two non-invasive and non-destructive photonic methods, UV-Vis spectroscopy and refractometry, to monitor infusions provided by syringe pumps. Refractometry was thereby applied to administered infusions supplemented by selected reference solutions. Although refractometry is generally not the first choice for identification of solutions, as the assignment is not unambiguous, we thus discovered that a clear assignment is possible for most of the infusions that are administered at specified (standardized) concentrations. The essential outcome of the refractometry measurements are:

- Eight out of forty-two solutions can be solely differentiated by the refractive index.
- The refractive index correlates linear with concentration for the applied infusions.
- Refractive index measurements are applicable over a broad range of concentrations.
- Low concentrations cannot be distinguished. The required LOD (20μg/mL) for catecholamines cannot be reached, as we found for norepinephrine 34μg/mL and epinephrine 25μg/mL.

Thus, refractometry alone is not able to differentiate all the applied infusions. These problems can predominantly be overcome by combining refractometry with spectrophotometry. The important results of the absorbance measurements are:

- Measurements are able to quantify the lowest concentration of the examined catecholamine infusions; LODs of norepinephrine and epinephrine are: 3μg/mL and 6μg/mL, respectively.
- Absorbance provides information about the molecular structure of the chromophore, enabling a broad classification of functional chemical groups.

In conclusion, by a combination of spectrometry and refractive index data, almost all infusions measured can be distinguished besides the catecholamines norepinephrine and epinephrine. These active agents are too similar in their chemical structure to be differentiated by these techniques. Moreover, the electrolytes NaCl 5.85% and KCl 7.45% cannot be discriminated by this combination. Furthermore, spectrophotometry and refractive index supplement each other very well considering the concentration range. Spectrophotometry works better at low concentrations while refractive index works also at high concentrations, at which the absorbance saturates.

This study is a first step in infusion monitoring concerning the multitude of infusions administered at intensive care units worldwide. However, an intelligent data fusion of the refractive index and the spectrophotometric data should be able to identify most infusions. The linear correlation of concentration with refractive index and absorbance, respectively, might be useful in generating an algorithm, which can identify infusions and their concentrations, being dosed according to the body weight of the individual patient.

Besides the specific assignment of the physical parameter to the medication, the sensor should correspond to the requirements of the intensive care units. These are packed with monitoring devices and various equipment. The increasing complexity of infusion therapy demands the simultaneous administration of up to twelve infusions by infusion pumps, sometimes exceeding twenty-four infusion pumps in a rack. Hence, a robust and compact technique is needed which suits the requirements of intensive care-units. Both refractive index and absorbance measurements are used outside of sophisticated analytical laboratories and hence are suited for point-of-care analytics. Finally, they can be operated in flow mode.

This study shows the potential and applicability of refractometry and spectrophotometry. At present, further infusions are measured and an algorithm for the data fusion is being developed, enabling the identification of infusions by a comparison with a data base. Finally, first efforts are carried out towards an implementation of the techniques in the intensive care unit. Therefore, a measuring cell is being constructed, which will be easily integrated in the workflow of the hospital staff members. The monitoring of infusions by the combination of the presented techniques is a promising approach to increase the patient safety in hospitals and facilitates the hospital staff member their work in intensive care units. In addition to the safety aspect, the device can also be used as a tool for creating detailed and automated documentation in hospitals.

## Figures and Tables

**Table 1:** List of solutions used and their respective refractive index.

Substances Used		
Composition of Solutions	Abbreviation	Refractive Index n
Glucose and Electrolyte Solutions		
Deionized water	DI-Water	1.33272
Aqua (pure water)	Aqua	1.33273
Glucose 5.00%	GLC 5.00%	1.33993
Glucose 10.0%	GLC 10.0%	1.34833
Glucose 15.0%	GLC 15.0%	1.35489
Glucose 20.0%	GLC 10.0%	1.36242
Glucose 25.0%	GLC 25.0%	1.36954
Glucose 30.0%	GLC 30.0%	1.37639
Glucose 35.0%	GLC 35.0%	1.38324
Glucose 40.0%	GLC 40.0%	1.39057
Sodium chloride 0.90%	NaCl 0.90%	1.33433
Sodium chloride 5.00%	NaCl 5.00%	1.34144
Sodium chloride 5.85% (1 M)	NaCl 5.85%	1.34270
Sodium chloride 10.0%	NaCl 10.0%	1.34933
Sodium chloride 15.0%	NaCl 15.0%	1.35822
Sodium chloride 20.0%	NaCl 20.0%	1.36486
Potassium chloride 5.00%	KCl 5.00%	1.33952
Potassium chloride 7.45% (1 M)	KCl 7.45%	1.34260
Potassium chloride 10.0%	KCl 10.0%	1.34664
Potassium chloride 15.0%	KCl 15.0%	1.35300
Potassium chloride 20.0%	KCl 20.0%	1.35917
Sodium bicarbonate 8.40%	NaBic 8.40%	1.34372

**Table 2:** List of the mixtures prepared and their respective refractive index.

Substances Used		
Composition of Solutions	Abbreviation	Refractive Index n
**Norepinephrine** **Solutions**		
5 mL Norepinephrine (5 mg)	Norepi 1000 μg/mL	1.33500
2.400 mL Norepinephrine + 0.600 mL DI-Water	Norepi 800 μg/mL	1.33450
1.800 mL Norepinephrine + 1.200 mL DI-Water	Norepi 600 μg/mL	1.33411
1.200 mL Norepinephrine + 1.800 mL DI-Water	Norepi 400 μg/mL	1.33369
0.600 mL Norepinephrine + 2.400 mL DI-Water	Norepi 200 μg/mL	1.33324
0.300 mL Norepinephrine + 2.700 mL DI-Water	Norepi 100 μg/mL	1.33297
0.240 mL Norepinephrine + 2.760 mL DI-Water	Norepi 80 μg/mL	1.33291
0.180 mL Norepinephrine + 2.820 mL DI-Water	Norepi 60 μg/mL	1.33286
0.120 mL Norepinephrine + 2.880 mL DI-Water	Norepi 40 μg/mL	1.33285
0.060 mL Norepinephrine + 2.940 mL DI-Water	Norepi 20 μg/mL	1.33277
0.030 mL Norepinephrine + 2.970 mL DI-Water	Norepi 10 μg/mL	1.33272
0.015 mL Norepinephrine + 2.985 mL DI-Water	Norepi 5 μg/mL	1.33273
0.003 mL Norepinephrine + 2.997 mL DI-Water	Norepi 1 μg/mL	1.33272
5.0 mL Norepinephrine (5 mg) + 45 mL GLC 5.00%	Norepi A	1.33943
5.0 mL Norepinephrine (5 mg) + 45 mL NaCl 0.90%	Norepi B	1.33437
5.0 mL Norepinephrine (5 mg) + 45 mL KCl 7.45%	Norepi C	1.34191
5.0 mL Norepinephrine (5 mg) + 45 mL GLC 10.0%	Norepi D	1.34607
**Epinephrine** **Solutions**		
5 mL Epinephrine (5 mg)	Epi 1000 μg/mL	1.33504
2.400 mL Epinephrine + 0.600 mL DI-Water	Epi 800 μg/mL	1.33449
1.800 mL Epinephrine + 1.200 mL DI-Water	Epi 600 μg/mL	1.33410
1.200 mL Epinephrine + 1.800 mL DI-Water	Epi 400 μg/mL	1.33365
0.600 mL Epinephrine + 2.400 mL DI-Water	Epi 200 μg/mL	1.33321
0.300 mL Epinephrine + 2.700 mL DI-Water	Epi 100 μg/mL	1.33298
0.240 mL Epinephrine + 2.760 mL DI-Water	Epi 80 μg/mL	1.33288
0.180 mL Epinephrine + 2.820 mL DI-Water	Epi 60 μg/mL	1.33285
0.120 mL Epinephrine + 2.880 mL DI-Water	Epi 40 μg/mL	1.33282
0.060 mL Epinephrine + 2.940 mL DI-Water	Epi 20 μg/mL	1.33277
0.030 mL Epinephrine + 2.970 mL DI-Water	Epi 10 μg/mL	1.33275
0.015 mL Epinephrine + 2.985 mL DI-Water	Epi 5 μg/mL	1.33273
0.003 mL Epinephrine + 2.997 mL DI-Water	Epi 1 μg/mL	1.33272
**Vancomycin** **Solutions**		
Vancomycin (500 mg) + 100 mL Aqua	VAN A	1.33367
Vancomycin + 10 mL Aqua + 90 mL GLC 5.00%	VAN B	1.34044
Vancomycin + 10 mL Aqua + 90 mL GLC 40.0%	VAN C	1.38540
Vancomycin + 5.0 mL Aqua + 45 mL GLC 5.00% +		
+ 20 mL NaCl 0.90%	VAN D	1.33858
Vancomycin + 10 mL Aqua + 90 mL GLC 40.0% +		
+ 20 mL NaCl 0.90%	VAN E	1.37095
Vancomycin + 5.0 mL Aqua + 45 mL GLC 5.00% +		
+ 20 mL NaCl 10%	VAN F	1.34312
Vancomycin + 10 mL Aqua + 90 mL GLC 5.00% +		
+ 20 mL KCl 7.45%	VAN G	1.34105
**Insulin** **Solutions**		
Insuman Rapid 40 I.U./mL	Insulin 40 I.U.	1.33579
Insuman Rapid 100 I.U./mL	Insulin 100 I.U.	1.33595

**Table 3:** Slope and intercept of the fits shown in Figure 1.

Compound	Intercept	Slope
Glucose (aq)	1.33320(28)	$1.44\left(1\right)\times 10^{-3}$
Potassium chloride (aq)	1.33288(23)	$1.33\left(2\right)\times 10^{-3}$
Sodium chloride (aq)	1.33305(30)	$1.63\left(3\right)\times 10^{-3}$

**Table 4:** Slope and intercept of the fits shown in Figure 3.

Compound	Intercept	Slope
Norepinephrine	1.33273(1)	$2.27\left(2\right)\times 10^{-6}$
Epinephrine	1.33272(1)	$2.28\left(2\right)\times 10^{-6}$

**Table 5:** Limit of detection (LOD) and limit of quantification (LOQ) for the catecholamine medications norepinephrine and epinephrine determined by refractometry within a concentration range of 0 to 1000μg/mL.

Compound	LOD [μg/mL]	LOQ [μg/mL]
Norepinephrine	33.7	109.2
Epinephrine	25.8	84.0

**Table 6:** Slope and intercept of the fits using either the peak height or the peak area, as shown in Figure 5, for norepinephrine.

Method	Intercept	Slope
Peak height	−0.0131(46)	0.00554(9)
Peak area	−0.0145(58)	0.0048(1)

**Table 7:** Limit of detection (LOD) and limit of quantification (LOQ) for catecholamine medications measured by UV-Vis absorbance.

Compound	LOD [μg/mL]	LOQ [μg/mL]
Norepinephrine	3.43	12.56
Epinephrine	6.31	22.72

## Data Availability

Not applicable.

## Supplementary Materials

The following supporting information can be downloaded at: https://www.mdpi.com/article/10.3390/diagnostics12040936/s1, Figure S1: Refractive index measurement at a wavelength of λ = 589 nm and a temperature of T = 23 °C for varying concentrations of norepinephrine and epinephrine within a concentration range between 0 and 100 μg/mL; Figure S2: **Top**: Normalized area of the main peak in the absorbance spectrum of norepinephrine for different concentrations. A saturation at higher concentrations is visible. Top right: Magnfication of the low-concentration region showing a linear behavior of the peak area with the concentration. **Bottom**: Normalized height and normalized peak area of the main absorption peak in comparison. Both methods yield a linear increase with increasing concentration (in the low-concentration-regime). For the slopes see main text.

## References

[1] Keers R.N., Williams S.D., Cooke J., Ashcroft D.M. (2013). Causes of medication administration errors in hospitals: A systematic review of quantitative and qualitative evidence. Drug Saf..

[2] Burns N., Alkaisy Z., Sharp E. (2018). Doctors attitudes towards medication errors at 2002 & 2015. Int. J. Health Care Qual. Assur..

[3] Callen J., McIntosh J., Li J. (2010). Accuracy of medication documentation in hospital discharge summaries: A retrospective analysis of medication transcription errors in manual and electronic discharge summaries. Int. J. Med. Inform..

[4] Dirik H.F., Samur M., Seren Intepeler S., Hewison A. (2019). Nurses’ identification and reporting of medication errors. J. Clin. Nurs..

[5] Fitzgerald R.J. (2009). Medication errors: The importance of an accurate drug history. Br. J. Clin. Pharmacol..

[6] Hakkarainen K.M., Hedna K., Petzold M., Hägg S. (2012). Percentage of patients with preventable adverse drug reactions and preventability of adverse drug reactions—A meta-analysis. PLoS ONE.

[7] Baniasadi S., Fahimi F., Shalviri G. (2008). Developing an adverse drug reaction reporting system at a teaching hospital. Basic Clin. Pharmacol. Toxicol..

[8] Davies E.C., Green C.F., Mottram D.R., Pirmohamed M. (2006). Adverse drug reactions in hospital in-patients: A pilot study. J. Clin. Pharm. Ther..

[9] Davies E.C., Green C.F., Taylor S., Williamson P.R., Mottram D.R., Pirmohamed M. (2009). Adverse drug reactions in hospital in-patients: A prospective analysis of 3695 patient-episodes. PLoS ONE.

[10] Dormann H., Neubert A., Criegee-Rieck M., Egger T., Radespiel-Tröger M., Azaz-Livshits T., Levy M., Brune K., Hahn E.G. (2004). Readmissions and adverse drug reactions in internal medicine: The economic impact. J. Intern. Med..

[11] Farcas A., Sinpetrean A., Mogosan C., Palage M., Vostinaru O., Bojita M., Dumitrascu D. (2010). Adverse drug reactions detected by stimulated spontaneous reporting in an internal medicine department in Romania. Eur. J. Intern. Med..

[12] Gholami K., Shalviri G. (1999). Factors associated with preventability, predictability, and severity of adverse drug reactions. Ann. Pharmacother..

[13] Pearson T.F., Pittman D.G., Longley J.M., Grapes Z.T., Vigliotti D.J., Mullis S.R. (1994). Factors associated with preventable adverse drug reactions. Am. J. Hosp. Pharm..

[14] Pourseyed S., Fattahi F., Pourpak Z., Gholami K., Shariatpanahi S.S., Moin A., Kazemnejad A., Moin M. (2009). Adverse drug reactions in patients in an Iranian department of internal medicine. Pharmacoepidemiol. Drug Saf..

[15] Summa-Sorgini C., Fernandes V., Lubchansky S., Mehta S., Hallett D., Bailie T., Lapinsky S.E., Burry L. (2012). Errors Associated with IV Infusions in Critical Care. Can. J. Hosp. Pharm..

[16] Escrivá Gracia J., Aparisi Sanz Á., Brage Serrano R., Fernández Garrido J. (2021). Medication errors and risk areas in a critical care unit. J. Adv. Nurs..

[17] Hartel M.J., Staub L.P., Röder C., Eggli S. (2011). High incidence of medication documentation errors in a Swiss university hospital due to the handwritten prescription process. BMC Health Serv. Res..

[18] Tubaishat A. (2019). The effect of electronic health records on patient safety: A qualitative exploratory study. Inform. Health Soc. Care.

[19] Valentin A., Capuzzo M., Guidet B., Moreno R., Metnitz B., Bauer P., Metnitz P. (2009). Errors in administration of parenteral drugs in intensive care units: Multinational prospective study. BMJ.

[20] MacFie C.C., Baudouin S.V., Messer P.B. (2016). An integrative review of drug errors in critical care. J. Intensive Care Soc..

[21] Makary M.A., Daniel M. (2016). Medical error—The third leading cause of death in the US. BMJ.

[22] Ohashi K., Dalleur O., Dykes P.C., Bates D.W. (2014). Benefits and risks of using smart pumps to reduce medication error rates: A systematic review. Drug Saf..

[23] Kirkendall E.S., Timmons K., Huth H., Walsh K., Melton K. (2020). Human-Based Errors Involving Smart Infusion Pumps: A Catalog of Error Types and Prevention Strategies. Drug Saf..

[24] Giuliano K.K. (2018). Intravenous Smart Pumps: Usability Issues, Intravenous Medication Administration Error, and Patient Safety. Crit. Care Nurs. Clin. N. Am..

[25] Young J., Slebodnik M., Sands L. (2010). Bar Code Technology and Medication Administration Error. J. Patient Saf..

[26] Giaquinto N., Scarpetta M., Ragolia M.A., Pappalardi P. Real-time drip infusion monitoring through a computer vision system. Proceedings of the 2020 IEEE International Symposium on Medical Measurements and Applications (MeMeA).

[27] Chen F.G., Wang J.Y., Chen S., Tu S.C., Chen K.Y. A Hang-and-Play Intravenous Infusion Monitoring System. Proceedings of the 2015 3rd International Conference on Applied Computing and Information Technology/2nd International Conference on Computational Science and Intelligence.

[28] Doesburg F., Oelen R., Renes M.H., Lourenço P.M., Touw D.J., Nijsten M.W. (2021). Multi-infusion with integrated multiple pressure sensing allows earlier detection of line occlusions. BMC Med. Inform. Decis. Mak..

[29] Bacon O., Hoffman L. (2020). System-Level Patient Safety Practices That Aim to Reduce Medication Errors Associated with Infusion Pumps: An Evidence Review. J. Patient Saf..

[30] Husch M., Sullivan C., Rooney D., Barnard C., Fotis M., Clarke J., Noskin G. (2005). Insights from the sharp end of intravenous medication errors: Implications for infusion pump technology. Qual. Saf. Health Care.

[31] Pahlow S., Weber K., Popp J., Wood B.R., Kochan K., Rüther A., Perez-Guaita D., Heraud P., Stone N., Dudgeon A. (2018). Application of Vibrational Spectroscopy and Imaging to Point-of-Care Medicine: A Review. Appl. Spectrosc..

[32] de Veij M., Vandenabeele P., de Beer T., Remon J.P., Moens L. (2009). Reference database of Raman spectra of pharmaceutical excipients. J. Raman Spectrosc..

[33] Xu K., Zhou R., Takei K., Hong M. (2019). Toward Flexible Surface-Enhanced Raman Scattering (SERS) Sensors for Point-of-Care Diagnostics. Adv. Sci..

[34] Farquharson S., Brouillette C., Smith W., Shende C. (2019). A Surface-Enhanced Raman Spectral Library of Important Drugs Associated With Point-of-Care and Field Applications. Front. Chem..

[35] Mayerhöfer T.G., Dabrowska A., Schwaighofer A., Lendl B., Popp J. (2020). Beyond Beer’s Law: Why the Index of Refraction Depends (Almost) Linearly on Concentration. Chemphyschem.

[36] Hang Y., Boryczka J., Wu N. (2022). Visible-light and near-infrared fluorescence and surface-enhanced Raman scattering point-of-care sensing and bio-imaging: A review. Chem. Soc. Rev..

[37] Chen H., Das A., Bi L., Choi N., Moon J.I., Wu Y., Park S., Choo J. (2020). Recent advances in surface-enhanced Raman scattering-based microdevices for point-of-care diagnosis of viruses and bacteria. Nanoscale.

[38] Wachs I.E., Roberts C.A. (2010). Monitoring surface metal oxide catalytic active sites with Raman spectroscopy. Chem. Soc. Rev..

[39] Gardiner D.J., Graves P.R. (1989). Practical Raman Spectroscopy.

[40] Alffenaar J.W.C., Jongedijk E.M., van Winkel C.A.J., Sariko M., Heysell S.K., Mpagama S., Touw D.J. (2021). A mobile microvolume UV/visible light spectrophotometer for the measurement of levofloxacin in saliva. J. Antimicrob. Chemother..

[41] Anthis N.J., Clore G.M. (2013). Sequence-specific determination of protein and peptide concentrations by absorbance at 205 nm. Protein Sci..

[42] Mayerhöfer T.G., Popp J. (2019). Beer’s Law—Why Absorbance Depends (Almost) Linearly on Concentration. Chem. Phys. Chem..

