# Supplementary material for: Exploration of an Alarm Sensor to Detect Infusion Failure Administered by Syringe Pumps
Source: Diagnostics (Basel). 2022 Apr 8;12(4):936. doi: 10.3390/diagnostics12040936 (PMC9032832; doi:10.3390/diagnostics12040936)
Supplement: Supplementary file 1 [file diagnostics-12-00936-s001.zip › diagnostics-1652506-supplementary.pdf]

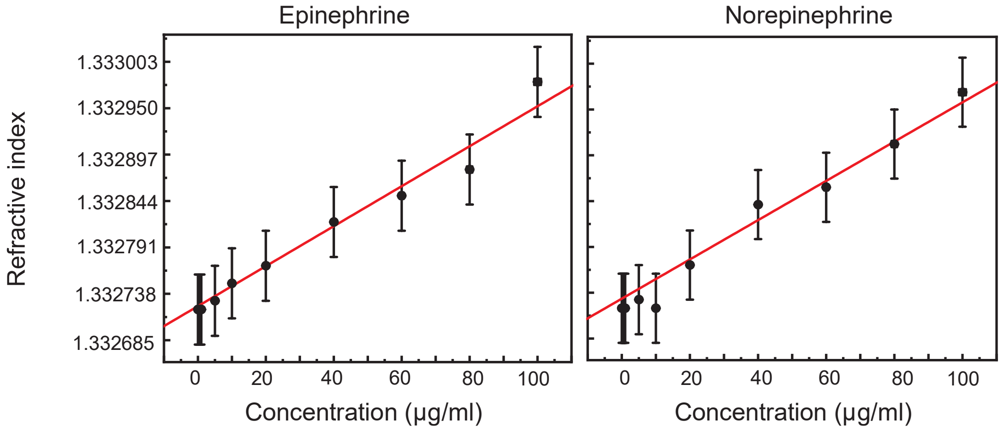

Figure S1. Refractive index measurement at a wavelength of $\lambda = 589$ nm and a temperature of T = 23 °C for varying concentrations of norepinephrine and epinephrine within a concentration range between 0 and 100 µg/ml.

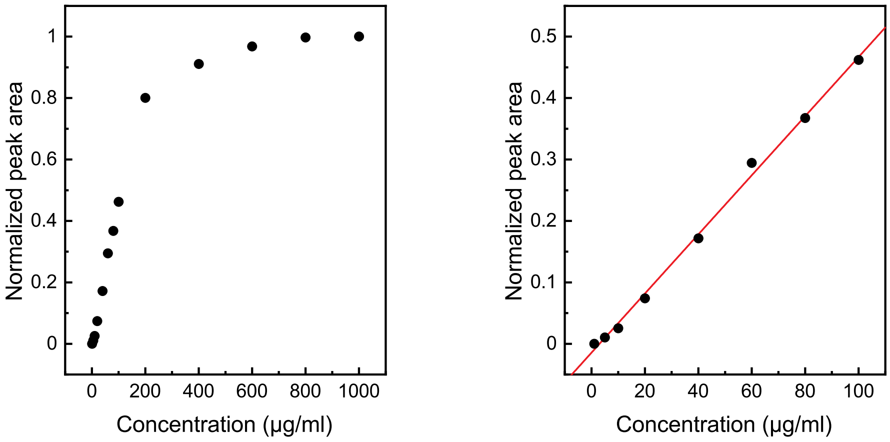

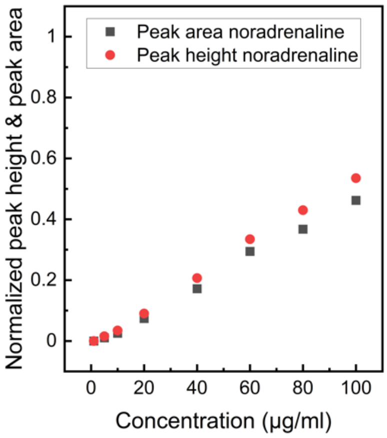

Figure S2: Top: Normalized area of the main peak in the absorbance spectrum of norepinephrine for different concentrations. A saturation at higher concentrations is visible. Top right: Magnfication of the low-concentration region showing a linear behavior of the peak area with the concentration. Bottom: Normalized height and normalized peak area of the main absorption peak in comparison. Both methods yield a linear increase with increasing concentration (in the low-concentration-regime). For the slopes see main text.
